# A Glucuronic Acid-Producing Endophyte *Pseudomonas* sp. MCS15 Reduces Cadmium Uptake in Rice by Inhibition of Ethylene Biosynthesis

**DOI:** 10.3389/fpls.2022.876545

**Published:** 2022-04-14

**Authors:** Lisheng Qian, Fei Song, Jinlin Xia, Rongfu Wang

**Affiliations:** ^1^College of Life Sciences, Anhui Agricultural University, Hefei, China; ^2^Anhui Shengnong Agricultural Group Co., Ltd., Maanshan, China

**Keywords:** endophytes, glucuronic acid, cadmium toxicity, ethylene, rice

## Abstract

Dynamic regulation of phytohormone levels is pivotal for plant adaptation to harmful conditions. It is increasingly evidenced that endophytic bacteria can regulate plant hormone levels to help their hosts counteract adverse effects imposed by abiotic and biotic stresses, but the mechanisms underlying the endophyte-induced stress resistance of plants remain largely elusive. In this study, a glucuronic acid-producing endophyte *Pseudomonas* sp. MCS15 alleviated cadmium (Cd) toxicity in rice plants. Inoculation with MCS15 significantly inhibited the expression of ethylene biosynthetic genes including *OsACO3*, *OsACO4*, *OsACO5*, *OsACS2,* and *OsACS5* and thus reduced the content of ethylene in rice roots. In addition, the expression of iron uptake-related genes including *OsIRT1*, *OsIRT2*, *OsNAS1*, *OsNAS2* and *OsYSL15* was significantly downregulated in the MCS15-inoculated roots under Cd stress. Similarly, glucuronic acid treatment also remarkably inhibited root uptake of Cd and reduced the production of ethylene. However, treatment with 1-aminocyclopropyl carboxylic acid (ACC), a precursor of ethylene, almost abolished the MCS15 or glucuronic acid-induced inhibition of Cd accumulation in rice plants. Conversely, treatment with aminoethoxyvinyl glycine (AVG), an inhibitor of ethylene biosynthesis, markedly reduced the Cd accumulation in plants. Taken together, our results revealed that the endophytic bacteria MCS15-secreted glucuronic acid inhibited the biosynthesis of ethylene and thus weakened iron uptake-related systems in rice roots, which contributed to preventing the Cd accumulation.

## Introduction

With the rapid development of industry and the intensive use of pesticides and chemical fertilizers, heavy metal contamination in soil has become increasingly serious (Sarwar et al., 2017). Cadmium (Cd) is one of the most common heavy metals that are highly toxic to living organisms, such as plants and animals (Dalcorso et al., 2013). Cd is readily taken up from the soil by plant roots and then enters into food chains, which pose a potential threat to animal and human health (Pan et al., 2019; Zhou et al., 2019). Many physical and chemical remediation strategies have been implemented for Cd-contaminated soils. In contrast, microbial remediation is a more eco-friendly and cost-effective method (Liu et al., 2018; Chen et al., 2019; Boros-Lajszner et al., 2021).

Deleterious effects of Cd stress on plants are attributable to serious interference with various physiological processes, such as inhibition of enzyme activities, overproduction of reactive oxygen species (ROS), and competition with other nutrients (Andresen and Küpper, 2013). The most affected nutrient is iron (Fe), which shares the similarities with Cd in chemical structure, behavior, soil availability, and nutrient uptake (Sebastian and Prasad, 2016; Zhou et al., 2019; Zhu et al., 2020). Excessive amounts of Cd in soil can lead to chlorosis of young leaves, which resemble the most typical symptom of Fe deficiency (Lešková et al., 2017). Cd stress provokes Fe deficient responses in plants due, at least in part, to the extensive substrate specificity of the Fe-regulated transporter 1(IRT1), which can transport divalent metals, such as zinc (Zn), nickel (Ni), and Cd (Clemens, 2006). In rice, several other metal transporters also participate in the mediation of Cd absorption and transport, including OsNramp1, OsNramp5, OsIRT1, and OsIRT2 (Nakanishi et al., 2006; Senoura et al., 2011; Sasaki et al., 2012). In *Arabidopsis*, the Cd-induced *IRT1* expression is positively associated with the enhanced Cd uptake, thereby exacerbating the Cd toxicity (Lei et al., 2020). Several hormones, such as abscisic acid (ABA), jasmonic acid (JA), and gibberellin (GA), can downregulate the expression of *IRT1* to inhibit root uptake of Cd, thus alleviating the Cd toxicity (Zhu et al., 2012; Pan et al., 2019; Lei et al., 2020). Therefore, the attenuated iron uptake-related systems may be a feasible route for blocking the Cd absorption in rice plants.

In Strategy I plants, Fe deficiency greatly stimulates root ethylene production (Romera and Alcantara, 2004; Wu et al., 2011). Upon exposure to 1-aminocyclopropyl carboxylic acid (ACC), a precursor of ethylene, the expression levels of Fe acquisition-related genes including transcription factors (*LeFER* and *AtFIT*), iron transporters (*AtIRT1*, *LeIRT1,* and *CsIRT1*), H^+^-ATPase (*CsHA1*), and ferric reductase oxidase 2 (*FRO2*) are greatly increased in many plant species, such as *Arabidopsis*, tomato, and cucumber plants. Conversely, treatment with the ethylene biosynthetic inhibitors, such as aminoethoxyvinyl glycine (AVG) and aminooxyacetic acid (AOA), largely weakens the transcription of these genes (Romera and Alcantara, 1994; Waters et al., 2007). Hence, Fe deficiency increases the production of ethylene, in turn, promotes the uptake of Fe regulated by *LeFER* or *AtFIT*. However, ethylene is not involved in the regulation of Fe deficient responses in Strategy II plants, except for rice that harbors two Fe acquisition (Strategy I and II) systems. In rice, Fe deficiency can induce the expression of genes encoding ACC synthases (ACS) and ACC oxidases (ACO), and increase ethylene levels (Zheng et al., 2009). Furthermore, ACC treatment alleviates Fe deficiency in rice by upregulating the expression of several iron uptake-related genes, such as *OsIRT1*, nicotianamine synthases 1 and 2 (*NAS1* and *NAS2*), and yellow-stripe like transporter 15 (*YSL15*; Wu et al., 2011). Increasing evidence indicates that ethylene regulates the expression of iron transporters that are also involved in Cd uptake in plants. Reduction of root ethylene production can effectively prevent Cd absorption in Strategy I plants (Ravanbakhsh et al., 2019). However, whether repression of ethylene biosynthesis hinders the Cd uptake in Strategy II plants, such as rice, is still lacking.

In nature, plants are colonized by countless soil microorganisms, which play important roles in maintaining plant health and productivity (Berendsen et al., 2012; Hardoim et al., 2015). Soil-borne bacteria have been demonstrated to regulate hormone homeostasis that helps host plants adapt to harmful environments (Etesami and Maheshwari, 2018; Eichmann et al., 2021). Microbe-mediated changes of plant hormone status are mainly due to microbial secretion of hormones, ACC deaminase-mediated decrease in ethylene synthesis, and release of volatile compounds (Ravanbakhsh et al., 2019; Abedini et al., 2021; Sharifi et al., 2022). Since it is difficult to monitor the physiological states of endophytes *in situ* and the complex effects of microbial metabolites on host hormone-associated pathways, the mechanisms of endophyte-inhibited Cd uptake in rice plants are not fully clear. In this study, we reported on the effect of the endophyte *Pseudomonas* sp. MCS15 on the Cd accumulation in rice plants. We further explored the mechanisms by which this bacterial strain improved the Cd resistance in rice plants by combining physiological and transcriptomic analyses.

## Materials and Methods

### Plant Materials, Growth Conditions, and Bacterial Inoculation

Rice (*Oryza sativa* L.) seeds were surface-sterilized for 15 min using 2.5% NaClO solution, followed by rinsing five times with sterile water. The sterilized seeds were placed in the darkness for 3 d and then transferred into hydroponic boxes containing sterile nutrient solution (1.43 mm Ca(NO_3_)_2_·4H_2_O, 1.43 mm (NH_4_)_2_SO_4_, 2.14 mm CaCl_2_, 1.34 mm KH_2_PO_4_, 1.02 mm K_2_SO_4_, 1.65 mm MgSO_4_·7H_2_O, 0.10 mm Na_2_SiO_3_·9H_2_O, 9.10 μm MnCl_2_·4H_2_O, 0.52 μm (NH_4_)_6_MO_7_O_24_·4H_2_O, 18.5 μm H_3_BO_3_, 0.15 μm ZnSO_4_·7H_2_O, 0.16 μm CuSO_4_·5H_2_O, and 35.8 μm EDTA-Fe). The hydroponic assays were performed in a growth chamber at 30°C/22°C (day/night) with a 16 h/10 h light/dark photoperiod.

Bacterial strains were isolated from root tissues of rice plants cultivated in Cd-contaminated soils (50 mg Cd kg^−1^ soil). About 1.0 g of root samples was sterilized with 2.5% NaClO solution for 10 min and then ground with sterile water and serially diluted and cultured on Luria-Bertani (LB) agar plates containing 100 μm CdCl_2_ at 28°C for 36 h. These bacterial isolates were purified and identified by 16S rRNA gene sequencing. For investigating the effects of a Cd-tolerant bacterium on the resistance of rice plants to Cd stress, *Pseudomonas* sp. MCS15 (GenBank No. OM678585) was initially incubated in LB liquid medium containing 5.0 g L^−1^ NaCl, 5.0 g L^−1^ yeast extract, and 10.0 g L^−1^ tryptone. Subsequently, bacterial suspensions were transferred into hydroponic boxes containing 1 l of sterile nutrient solution with or without 80 μm CdCl_2_. Each box harbored 16 seedlings. These boxes were divided into four treatments: the boxes without CdCl_2_ and MCS15 (−Cd), the boxes without CdCl_2_ and with MCS15 (−Cd + MCS15), the boxes with CdCl_2_ and without MCS15 (+Cd), and the boxes with CdCl_2_ and MCS15 (+Cd + MCS15), each treatment was conducted in triplicates. In the groups with MCS15, bacterial suspensions were poured into each box to a final concentration of 2 × 10^7^ CFU ml^−1^. The culture solutions were refreshed every 2 days.

### Measurement of Chlorophyll Content and Ethylene Production

Total chlorophyll content was estimated according to the method reported by Fan et al. (2014). Briefly, about 0.5 g of fresh leaves was immersed in 20 ml of 80% (v/v) acetone and then kept in the darkness at room temperature. After 24 h of incubation, the extract solutions were centrifuged at 12,000 *g* for 10 min. Total chlorophyll content was determined by measuring the absorbance of the extracted solutions at 645 and 663 nm according to the formula: 20.21 × A645 + 8.02 × A663 (Porra, 2002).

The production of ethylene in rice roots was measured according to the method described by Wu et al. (2011). Briefly, detached rice roots were transferred into 10 ml glass vials containing 1 ml of sterile water and then immediately sealed. These vials were cultured in the darkness for 5 h at 30°C, and 1 ml of headspace gas was then injected into a gas chromatography (Focus GC, Thermo, United States) for analyzing ethylene content. Total amount of ethylene was calculated according to fresh weight (FW) of root samples and expressed as nL ethylene g^−1^ FW h^−1^.

### Determination of Cd Content in Rice Tissues

Total amount of Cd in plant tissues was analyzed according to the method reported by Ding et al. (2018). Briefly, shoot and root samples were subjected to successive rinse with sterile deionized water, 5 mm CaCl_2_ and 5 mm EDTA and then dried overnight at 80°C. After that, 0.5 g of plant tissues was digested with HNO_3_/HClO_4_ (4:1, v/v) at 180°C for 3 h. The digested solution was diluted with deionized water for determining total amount of Cd. The concentrations Cd in plant tissues were detected using an atomic absorption spectrometer.

### Screening of ACC Deaminase Enzyme Activity

*Pseudomonas* sp. MCS15 was cultured in LB liquid medium for 24 h and then centrifuged at 8000 *g* for 10 min. The precipitate was washed and resuspended in sterile water. Subsequently, the bacterial suspensions were diluted and cultured on ADF medium amended with 3 mm ACC instead of (NH_4_)_2_SO_4_ according to Penrose and Glick (2003). These plates were incubated at 28°C for 3 d, and growing bacterial colonies were inferred as the ACC deaminase-producing bacteria.

### Transcriptome and qPCR Analyses

Total RNA was extracted from rice roots subjected to different treatments using TRIzol reagent (Takara, Japan) according to the manufacturer’s instructions. An Agilent 2,100 Bioanalyzer was then used to detect the concentration and purity of the extracted RNA samples. Construction of cDNA libraries from different treatments was performed using TruSeq RNA Sample Prep Kit v2 (Illumina, San Diego, United States) according to Yue et al. (2021). RNA sequencing (RNA-Seq) was conducted using the Illumina HiSeq 4,000 platform by the Biomarker Technologies Corporation (Beijing, China). Three biological repeats were conducted for RNA-Seq. Raw sequencing data were deposited in NCBI SRA database (accession No. PRJN807004). High-quality clean reads were obtained by removal of adapter and low-quality reads from the resulting raw data. DESeq2 software was applied to analyze differentially expressed genes at adjusted *value of p* < 0.05 and |log_2_ (fold change) > 1.5|. These DEGs were further used for gene ontology (GO) enrichment analyses by Goatools software (Tang et al., 2018).

For qPCR analysis, total RNA samples were reversely transcripted into cDNA with the PrimeScript Reverse RT reagent kit (Takara, Japan) according to the manufacturer’s protocols. qPCR reactions were carried out on an ABI Model 7,500 machine using the SYBR Green qPCR Master Mix (Takara, Japan) according to Zhu et al. (2021), as follows: 30 s at 94°C; 15 s at 95°C, 30 s at 60°C for 40 cycles. All the reactions were performed in three biological repeats. The rice *ACTIN1* gene was used as an internal reference. Relative expression levels of targeted genes were calculated using the 2^−ΔΔCt^ method (Livak and Schmittgen, 2001). In addition, standard curves for quantifying the 16S rRNA gene copy number of MCS15 were estimated by qPCR according to the method reported by Cotto et al. (2015). The primers used for qPCR analyses were listed in Supplementary Table S1.

### Measurement of Glucuronic Acid in Bacterial Cultures and Rice Roots

To analyze the production of glucuronic acid in bacterial cultures, *Pseudomonas* sp. MCS15 was firstly cultured in LB liquid medium at 28°C for different times. After that, the bacterial culture was centrifuged at 12,000 *g* at 4°C for 10 min. Finally, the supernatant was filtered by 0.22 μm microporous membrane for high performance liquid chromatography (HPLC). In addition, to measure the content of glucuronic acid in rice plants, root tissues were ground with liquid nitrogen and mixed with 70% MeOH and 1.0% formic acid. The extracted solution was incubated for at 4°C 30 min, followed by centrifugation at 12,000 g and 4°C for 10 min. Then, the levels of glucuronic acid were determined in the supernatant by HPLC. Agilent 1,260 chromatograph equipped with Venusil HILIC (250 mm × 4.6 mm, 5 μm) was used for detecting the content of glucuronic acid. The column temperature was kept at 35°C; 4 mm NaH_2_PO_4_ was used as the mobile phase; the flow rate was 0.8 ml min^−1^; the ultraviolet detection wavelength was 210 nm.

### Statistical Analysis

Data were statistically analyzed using SPSS 18.0 software. Significant difference among different treatments was analyzed using Tukey’s multiple comparison test at *p* < 0.05. All the experiments were performed at least three replicates for each treatment.

## Results

### Inoculation of Rice Plants With MCS15 Alleviated Cd Toxicity

**Figure 1:**
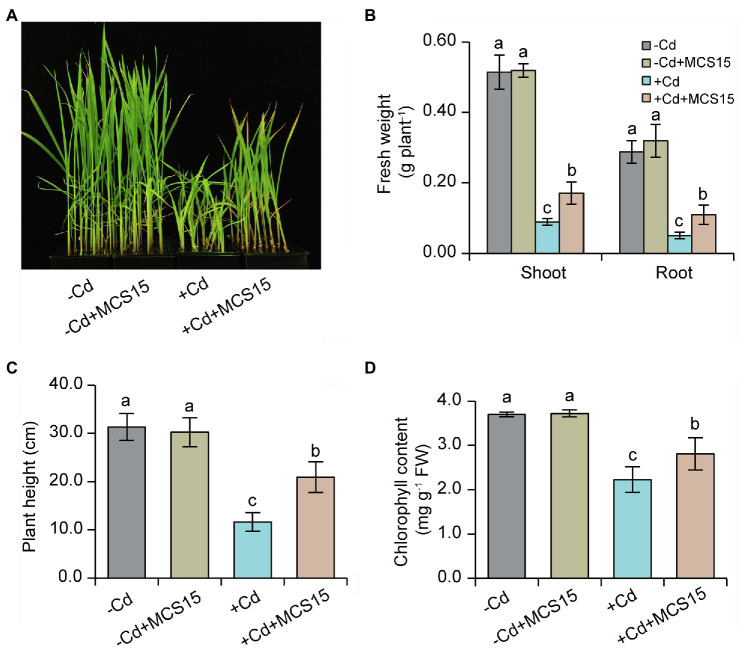
Effects of *Pseudomonas* sp. MCS15 on Cd resistance in rice plants. Rice seedlings were initially cultured in nutrient medium for 10 d and then transferred to the medium with either 80 μm CdCl_2_, bacterial suspension of MCS15, or both. **(A)** Plant phenotypes, **(B)** shoot and root fresh weights, **(C)** plant height, and **(D)** leaf chlorophyll content were determined after 2 weeks of treatments. Different letters indicate significant differences using Tukey’s multiple comparison test at *p* < 0.05. Error bars show ± SD from *n* = 3 biological repeats. Treatments: −Cd, 0 μm CdCl_2_; −Cd + MCS15, 0 μm CdCl_2_ and MCS15; +Cd, 80 μm CdCl_2_; +Cd + MCS15, 80 μm CdCl_2_ and MCS15.

**Figure 2:**
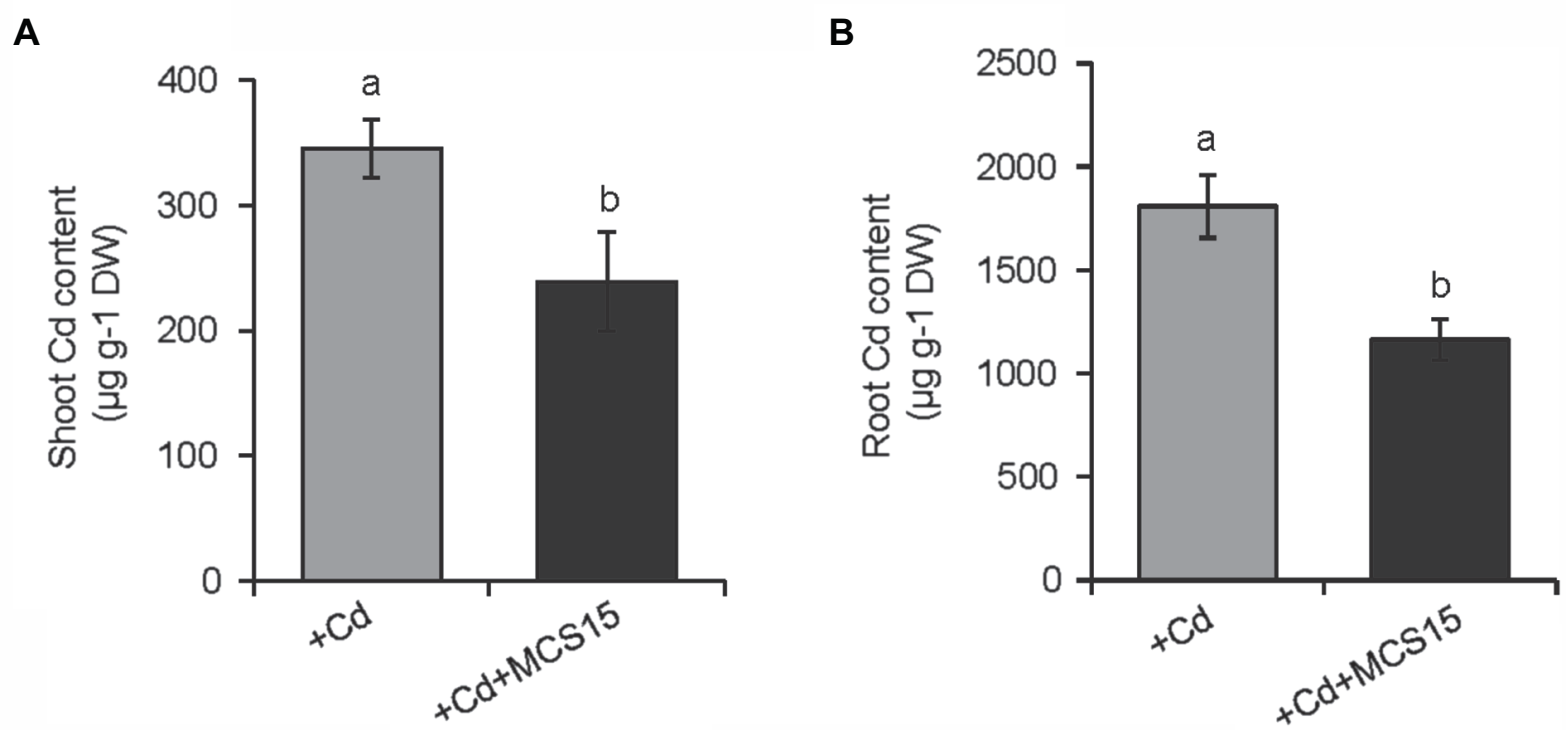
Effects of *Pseudomonas* sp. MCS15 on Cd accumulation in rice plants. Rice seedlings were initially cultured in nutrient medium for 10 d and then transferred to the medium with either 80 μm CdCl_2_, bacterial suspension of MCS15, or both. Different letters indicate significant differences using Tukey’s multiple comparison test at *p <* 0.05. Error bars show ± SD from *n* = 3 biological repeats. **(A)** Shoot and **(B)** root Cd content were determined after 2 weeks of treatments. Treatments: +Cd, 80 μm CdCl_2_; +Cd + MCS15, 80 μm CdCl_2_ and MCS15.

To examine the effects of *Pseudomonas* sp. MCS15 on Cd resistance in rice plants, 10-d-old rice seedlings were transplanted into the medium supplemented with 80 μm CdCl_2_ in the presence or absence of MCS15. As shown in Figure 1A, after 2 weeks of co-culture, MCS15 had no marked effects on rice growth under non-Cd stress. Plant growth was greatly inhibited by Cd stress, whereas fresh weights and plant height of the inoculated plants were markedly higher than those of the non-inoculated (control) plants (Figures 1B,C). Under Cd stress condition, the chlorophyll content of the control plants was remarkably decreased, while the inoculated plants remained relatively higher chlorophyll content (Figure 1D). After 2 weeks of Cd treatment, the content of Cd was significantly increased in shoots and roots of rice plants. Compared with Cd treatment alone, the inoculation of rice plants with MCS15 reduced the shoot and root Cd content by 29.2 and 39.8%, respectively (Figures 2A,B). Additionally, the colonization of MCS15 in the roots was quantified by qPCR analyses. The population of MCS15 was remarkably increased (5.7 × 10^6^ CFU g^−1^) after 2 weeks of co-culture. In contrast, the bacterial colonization was observably higher in the non-Cd-treated roots than that in the Cd-treated roots (Supplementary Figure S1).

### Transcriptome Analysis of MCS15-Inoculated Rice Roots Under Cd Stress

**Figure 3:**
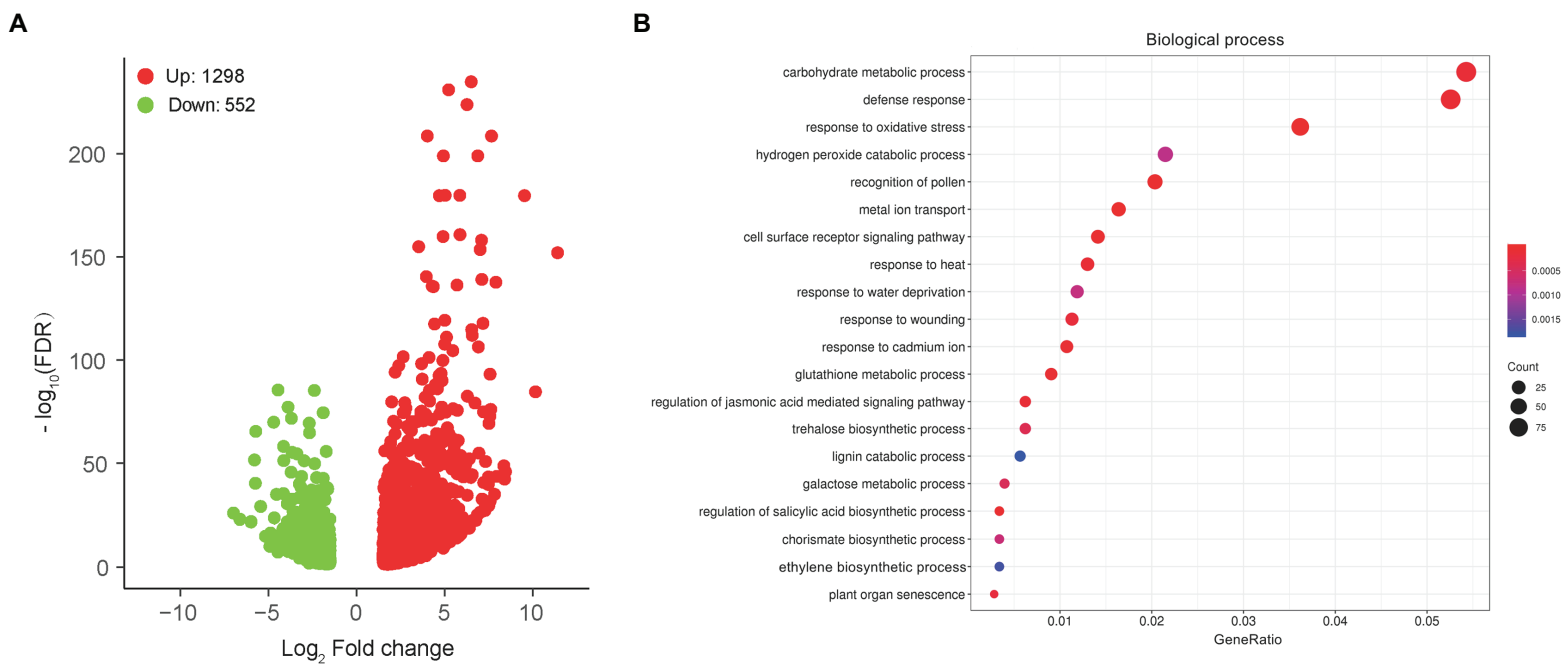
Transcriptome analyses of *Pseudomonas* sp. MCS15-inoculated rice roots under Cd stress. Rice seedlings were initially cultured in nutrient medium for 10 d and then transferred to the medium with either 80 μm CdCl_2_, bacterial suspension of MCS15, or both. After 24 h of treatments, root tissues from the non-inoculated (control) and MCS15-inoculated plants were collected for RNA-Seq analyses. **(A)** Volcano plot of DEGs between the control and inoculated plants. **(B)** GO enrichment analysis for these DEGs.

To unravel the molecular mechanisms of the MCS15-induced Cd resistance in rice plants, whole gene expression profiles in the Cd-treated roots were examined by RNA-Seq for identifying responsive genes to MCS15. For this purpose, 10-d-old rice seedlings were treated with 80 μm CdCl_2_ in the presence or absence of MCS15 for 24 h. After that, we analyzed differentially expressed genes in the roots between the non-inoculated (control) and inoculated plants. A total of 1,168 genes were differentially expressed in the roots with or without the bacterial inoculation under the thresholds of FDR < 0.05 and |log_2_FC| = ≥1.5. Among these DEGs, 614 genes were upregulated and 554 genes were downregulated (Figure 3A; Supplementary Table S2).

GO enrichment analysis for aforementioned DEGs showed that top 20 GO term annotations in the biological process (BP) category were associated with multiple processes, such as “carbohydrate metabolic process,” “defense response,” “response to oxidative stress,” “metal ion transport,” and “ethylene biosynthetic stress” (Figure 3B). In the “metal ion transport” pathway, a total of 5 DEGs associated with iron uptake and transport were significantly downregulated, including *OsIRT1*, *OsIRT2*, *OsNAS1*, *OsNAS2,* and *OsYSL15*. Furthermore, “ethylene biosynthetic process” was the pathway that included a total of 6 DEGs (1 upregulation and 5 downregulation), in which the expression of *OsACO1* was upregulated, whereas the expression of *OsACS2*, *OsACS5*, *OsACO3*, *OsACO4,* and *OsACO5* was downregulated (Supplementary Table S3).

### Inhibition of Ethylene Biosynthesis by MCS15 Led to Reduction of Cd Content in Rice

**Figure 4:**
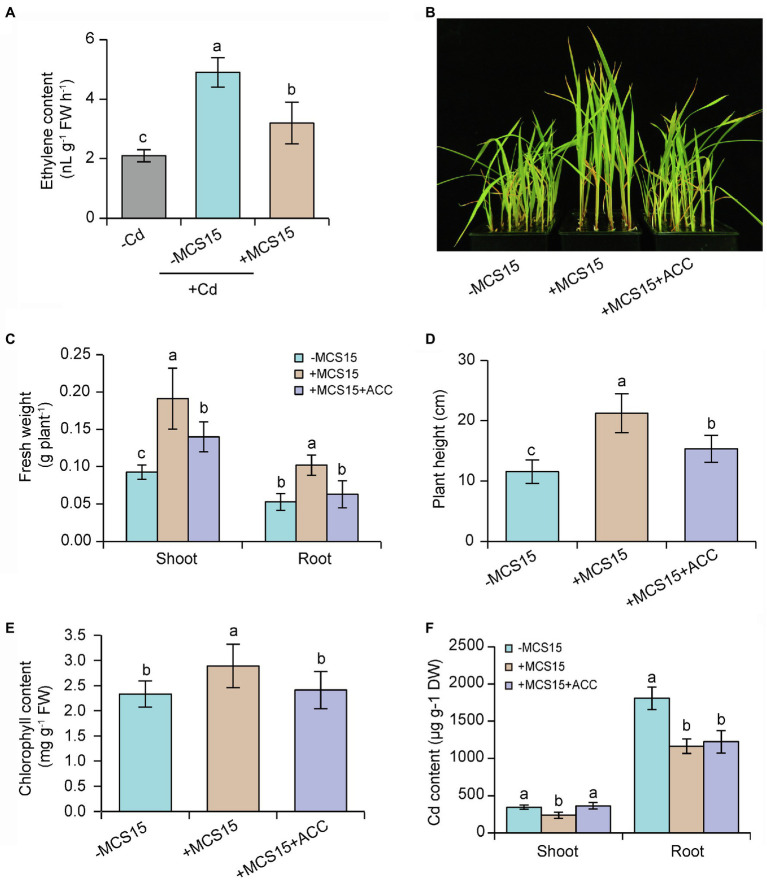
Effects of ethylene on the *Pseudomonas* sp. MCS15-induced Cd resistance of rice plants. Rice seedlings were initially cultured in nutrient medium for 10 d and then transferred to the medium containing 80 μm CdCl_2_ with or without the addition of MCS15 and/or 50 μm ACC. **(A)** Root ethylene production, **(B)** plant phenotypes, **(C)** shoot and root fresh weight, **(D)** plant height, **(E)** chlorophyll content, **(F)** shoot and root Cd content were determined after 2 weeks of treatments. Different letters indicate significant differences using Tukey’s multiple comparison test at *p <* 0.05. Error bars show ± SD from *n* = 3 biological repeats. Treatments: −Cd, 0 μm CdCl_2_; +Cd, 80 μm CdCl_2_; −MCS15, the medium without MCS15; +MCS15, the medium with MCS15; +MCS15 + ACC, the medium with MCS15 and 50 μm ACC.

Ethylene can activate the transcription of iron uptake-related genes in rice plants, but high-level expression of these genes enhances Cd uptake and thereby aggravates the Cd toxicity (Ravanbakhsh et al., 2019). It was thus possible that the MCS15-induced Cd resistance in rice plants was attributable to the inhibited ethylene biosynthesis. In this study, 10-d-old rice seedlings were treated with 80 μm CdCl_2_ in the presence or absence of MCS15 for 3 d. As shown in Figure 4A, root ethylene levels were significantly increased in the Cd-treated plants compared with the un-treated plants, whereas the content of ethylene in the roots was markedly less in the MCS15-inoculated plants than the non-inoculated plants. Furthermore, the ACC deaminase enzyme activity of MCS15 was evaluated in the ADF medium amended with 3 mM ACC as sole nitrogen source. The bacterial strain was not able to grow on the medium, which indicated that MCS15 was not an ACC deaminase producer.

**Figure 5:**
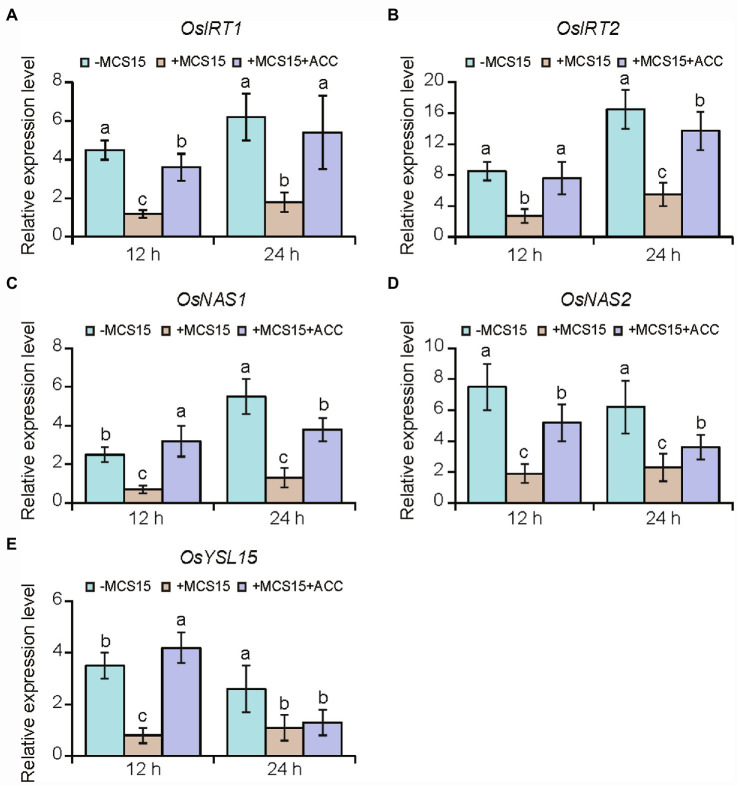
Effects of ethylene on the expression of iron uptake-related genes in the roots of *Pseudomonas* sp. MCS15-inoculated rice plants. Rice seedlings were initially cultured in nutrient medium for 10 d and then transferred to the medium containing 80 μm CdCl_2_ with or without the addition of MCS15 and/or 50 μm ACC. **(A)** *OsIRT1*, **(B)** *OsIRT2*, **(C)** *OsNAS1*, **(D)** *OsNAS2*, **(E)** *OsYSL15* were determined after 12 and 24 h of treatments, respectively. Different letters indicate significant differences using Tukey’s multiple comparison test at *p <* 0.05. Error bars show ± SD from *n* = 3 biological repeats. Treatments: −Cd, 0 μm CdCl_2_; +Cd, 80 μm CdCl_2_; −MCS15, the medium without MCS15; +MCS15, the medium with MCS15; +MCS15 + ACC, the medium with MCS15 and 50 μm ACC.

To confirm the hypothesis that ethylene was involved in the MCS15-induced Cd resistance in rice plants, we evaluated the impacts of MCS15 on the Cd accumulation in rice plants treated with 50 μm ACC, an ethylene biosynthetic precursor. As shown in Figure 4B, ACC treatment abrogated the MCS15-alleviated Cd toxicity in rice plants, as reflected by reduction of biomass, plant height, and chlorophyll content (Figures 4C–E). Consistently, the MCS15-induced inhibition of Cd uptake was remarkably weakened by ACC treatment (Figure 4F). Furthermore, the expression of iron uptake-related genes including *OsIRT1*, *OsIRT2*, *OsNAS1*, *OsNAS2,* and *OsYSL15* was increased by 2.3–15.8 folds in the Cd-treated roots, while their expression levels were reduced by 1.7–4.8 folds in the MCS15-inoculated roots. However, the addition of ACC to the medium abolished the MCS15-induced effects (Figures 5A–E). Hence, the inhibited ethylene-mediated activation of iron uptake-related systems contributed to alleviating Cd toxicity in rice plants.

### MCS15-Secreted Glucuronic Acid Was Involved in Mediating Root Ethylene Production

**Figure 6:**
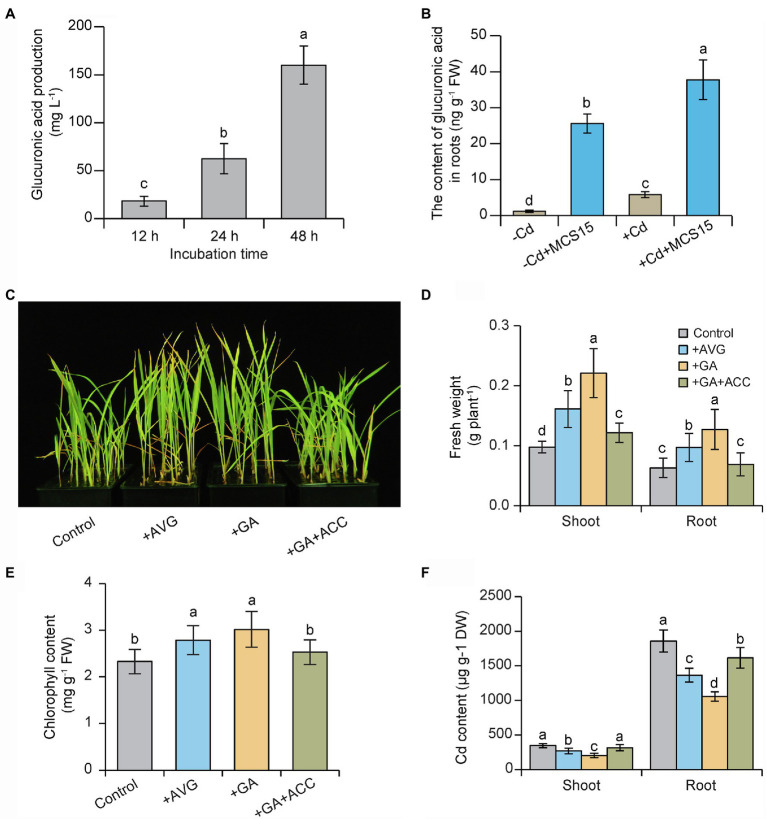
Effects of glucuronic acid on Cd resistance of rice plants. **(A)** The production of glucuronic acid in bacterial cultures of MCS15. **(B)** 10-d-old rice seedlings were treated with 0 or 80 μm CdCl_2_ in the presence or absence of MCS15 for 24 h. Then, the content of glucuronic acid in the roots was determined. In addition, rice seedlings were initially cultured in nutrient medium for 10 d and then transferred to the medium containing 80 μm CdCl_2_ with or without the presence of 20 μm AVG, 1 mm glucuronic acid, co-treatment of 1 mm glucuronic acid, and 50 μm ACC. **(C)** Plant phenotypes, **(D)** shoot and root fresh weights, **(E)** chlorophyll content, and **(F)** shoot and root Cd content were determined after 2 weeks of treatments. Different letters indicate significant differences using Tukey’s multiple comparison test at *p <* 0.05. Error bars show ± SD from *n* = 3 biological repeats. Treatments: −Cd, 0 μm CdCl_2_; +Cd, 80 μm CdCl_2_; −MCS15, the medium without MCS15; +MCS15, the medium with MCS15; +MCS15 + ACC, the medium with MCS15 and 50 μm ACC.

In this study, HPLC analysis revealed that the production of glucuronic acid was gradually increased in bacterial cultures of MCS15 followed by prolonged incubation times (Figure 6A), which indicated that MCS15 was a glucuronic acid-producing bacterium. Consistently, the MCS15-inoculated roots accumulated higher levels of glucuronic acid than the controls under non-Cd and Cd stress conditions (Figure 6B). The effects of glucuronic acid treatment on plant response to Cd stress were further examined. As shown in Figure 6C, glucuronic acid treatment markedly increased Cd resistance in rice plants, as reflected by higher fresh weight and chlorophyll content (Figures 6D,E). Furthermore, shoot and root Cd content was significantly less in the glucuronic acid-treated plants than the un-treated (control) plants (Figure 6F).

**Figure 7:**
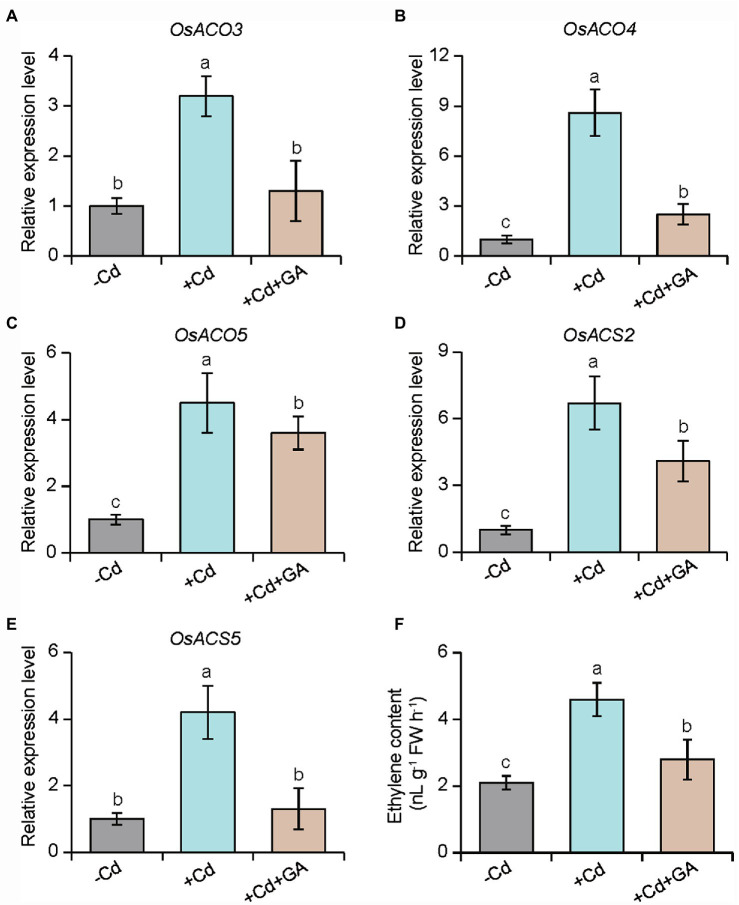
Effects of glucuronic acid on the expression of several ethylene biosynthetic genes in rice plants. Rice seedlings were initially cultured in nutrient medium for 10 d and then transferred to the medium either 80 μm CdCl_2_, 1 mm glucuronic acid, or both. The expression of **(A)** *OsACO3*, **(B)** *OsACO4*, **(C)** *OsACO5*, **(D)** *OsACS2,* and **(E)** *OsACS5*, and **(F)** ethylene content were determined in rice roots after 2 weeks of treatments. Different letters indicate significant differences using Tukey’s multiple comparison test at *p <* 0.05. Error bars show ± SD from *n* = 3 biological repeats. Treatments: −Cd, 0 μm CdCl_2_; +Cd, 80 μm CdCl_2_; +Cd + GA, 80 μm CdCl_2_ and 1 mm glucuronic acid.

We further tested the effects of ethylene treatment on the glucuronic acid-induced Cd resistance in rice plants. As shown in Figures 6A–C, the glucuronic acid-induced Cd resistance in rice plants was largely weakened by ACC treatment. Conversely, treatment with an ethylene biosynthetic inhibitor, AVG, distinctly enhanced the resistance of rice plants to Cd stress. Similar results were observed for the content of Cd in the rice plants subjected to AVG or ACC treatment (Figure 6D). Furthermore, the expression of ethylene biosynthetic genes including *OsACO3*, *OsACO4*, *OsACO5*, *OsACS2,* and *OsACS5* was increased by 2.1–8.6 folds in the Cd-treated roots, but their expression levels were reduced by 1.2–4.3 folds in the glucuronic acid-treated roots (Figures 7A–E). This result was consistent with reduction of ethylene content in the glucuronic acid-treated plants under Cd stress (Figure 7F).

## Discussion

Heavy metal pollution is a commonly existing problem in farmland, which threatens food safety (Rai et al., 2019; Qin et al., 2021). Due to the difficulty and high cost of soil decontamination, it is urgently needed to develop alternative strategies for dealing with heavy metal pollution in agroecosystems (Kavamura and Esposito, 2010; Wan et al., 2016). Manipulation of plant physiology to prevent the accumulation of heavy metals may help to grow safe food on contaminated soil (Wu et al., 2018; Ravanbakhsh et al., 2019; Han et al., 2020; Tanwir et al., 2021). In this study, we examined whether endophytic bacteria could help limit plant uptake of Cd by regulating hormone balance. It was found that the secretion of glucuronic acid by the endophytic bacteria MCS15 inhibited the expression of ethylene-mediated activation of iron uptake-related genes in rice roots, thereby reducing the Cd accumulation.

In plants, ethylene is a central hormone, which plays a pivotal role in regulation of stress adaptation and can be used as an effective target to control the absorption of heavy metals (Verma et al., 2016; Ravanbakhsh et al., 2019). Ethylene regulates multiple plant traits related to stress responses, but the same stress response beneficial to plants may make it harmful: several metal ion transporters, such as Fe^2+^ and Zn^2+^, can be induced by ethylene, which confers the increased accumulation of heavy metals (Ravanbakhsh et al., 2019). It is increasingly aware that the levels of ethylene in plants can be steered by microorganisms that degrade or increase the levels of this hormone (Ravanbakhsh et al., 2019; Sun et al., 2022). It is well documented that ACC deaminase-producing bacteria can change plant physiology, which is similar to the mutants defective in ethylene signal transduction pathways (Santoyo et al., 2016). ACC, the precursor of ethylene biosynthesis can be converted into α-ketobutyrate and ammonia by bacterial ACC deaminase, thereby mediating the levels of endogenous ethylene in plants under adverse stresses (Glick, 2005; Jin et al., 2015; Bouffaud et al., 2018; Naing et al., 2021). The ACC deaminase-producing bacteria can enhance the tolerance of plants to various stresses, such as drought, salt, and waterlogging, by inhibiting overproduction of ethylene (Ali and Kim, 2018; Gupta and Pandey, 2019; Orozco-Mosqueda et al., 2019; Danish et al., 2021). The ACC deaminase-producing bacteria have also been reported to mediate the production of ethylene and thus alleviate Cd stress by inhibiting the expression of Fe acquisition-related genes, such as *HMA3* and *Nramp5* (Sun et al., 2022). In this study, under Cd stress, a large number of DEG in the roots of rice seedlings treated with MCS15 were enriched to the “ethylene synthesis” and “metal ion transport” pathways, indicating that MCS15 may help to reduce the Cd toxicity in rice plants through these pathways. To investigate the roles of ethylene in mediating the accumulation of Cd in rice plants, we conducted hydroponic experiments on the Cd-treated rice plants treated with AVG, an ethylene biosynthetic inhibitor. Consistent with the effect of MCS15 on the Cd absorption in rice, the accumulation of Cd in the AVG-treated plants was significantly lower than those in the un-treated plants, confirming the role of ethylene in mediating the Cd absorption in rice plants. In addition, treatment with ACC abolished the MCS15-induced effects, indicating that steering ethylene metabolism was essential for the MCS15-alleviated Cd toxicity in rice plants. However, this bacterial strain did not show any ACC deaminase activity. Therefore, the other mechanisms had been adopted by MCS15 to operate host ethylene production under Cd stress.

Ample evidence has indicated that ethylene has a positive impact on several pathways involved in metal detoxification, including metal chelation and transport to the aboveground parts of plants (Arshad et al., 2007; Zhang et al., 2014; Thao et al., 2015; Ravanbakhsh et al., 2019). These mechanisms are important for heavy metal tolerance, but may confer the increased heavy metal accumulation in aboveground parts, resulting in food safety problems. The regulation of heavy metal transporters is an important mechanism to reduce the toxicity of heavy metals in plants (Nakanishi et al., 2006; Zhu et al., 2012). Although there are no specific Cd transporters, several metal transporters, such as OsIRT1 and OsIRT2, are considered to be involved the transport of Cd in addition to the transport of other divalent metal ions, such as Fe^2+^, Zn^2+^, and Mn^2+^ (Ishimaru et al., 2006; Nakanishi et al., 2006; Li et al., 2019). It is recently reported that Cd exposure increases the expression levels of *OsNramp1*, *OsNramp5*, *OsIRT1,* and *OsIRT2* in rice roots (Jiang et al., 2020; Zhang et al., 2022). Similarly, Cd stress increased the expression of several iron uptake-related genes, such as *OsNAS1*, *OsNAS2*, *OsYSL15*, *OsIRT1,* and *OsIRT2,* in rice roots. However, after exposure to MCS15, the expression of these iron uptake-related transporters was decreased significantly, indicating that MCS15 inhibited the expression of these genes induced by Cd stress. ACC treatment reversed the increased expression of these transporter genes, which may be the main reason for the MCS15-induced Cd resistance in rice plants. In this study, MCS15 was a glucuronic acid-producing endophytic bacterium and thus led to higher levels of glucuronic acid in the rice roots. Many studies have shown that high-level organic acids can improve the tolerance of plants to adverse stresses (Hawrylak-Nowak et al., 2015; Kim et al., 2017; Utsumi et al., 2019). Acetic acid can improve drought tolerance in many plant species, such as cassava and *Arabidopsis* by activation of ABA or JA signaling pathways (Utsumi et al., 2019). Treatment with organic acids, such as citric acid, tartaric acid, and malic acid, can alleviate structural damages in the photosynthetic apparatus imposed by Cd stress (Chen et al., 2020). In addition, exogenous malic and acetic acids have also been reported to ameliorate the Cd toxicity in sunflower plants (Hawrylak-Nowak et al., 2015). Hence, higher levels of glucuronic acid in the MCS15-inoculated roots likely contributed to relieving the Cd toxicity in rice plants. To verify this hypothesis, we examined the impacts of glucuronic acid on the Cd-tolerating capacity of rice plants. Glucuronic acid treatment distinctly enhanced the resistance of rice plants to Cd stress, but failed to mitigate the Cd toxicity in the ACC-exposed rice plants. Furthermore, the expression of several ethylene biosynthetic genes in the Cd-treated roots was remarkably inhibited by glucuronic acid. These results strongly supported a pivotal role of exogenous glucuronic acid supplementation as well as the glucuronic acid-producing MCS15 endophytic bacterial inoculation induced the tolerance to Cd stress in rice plants.

## Conclusion

**Figure 8:**
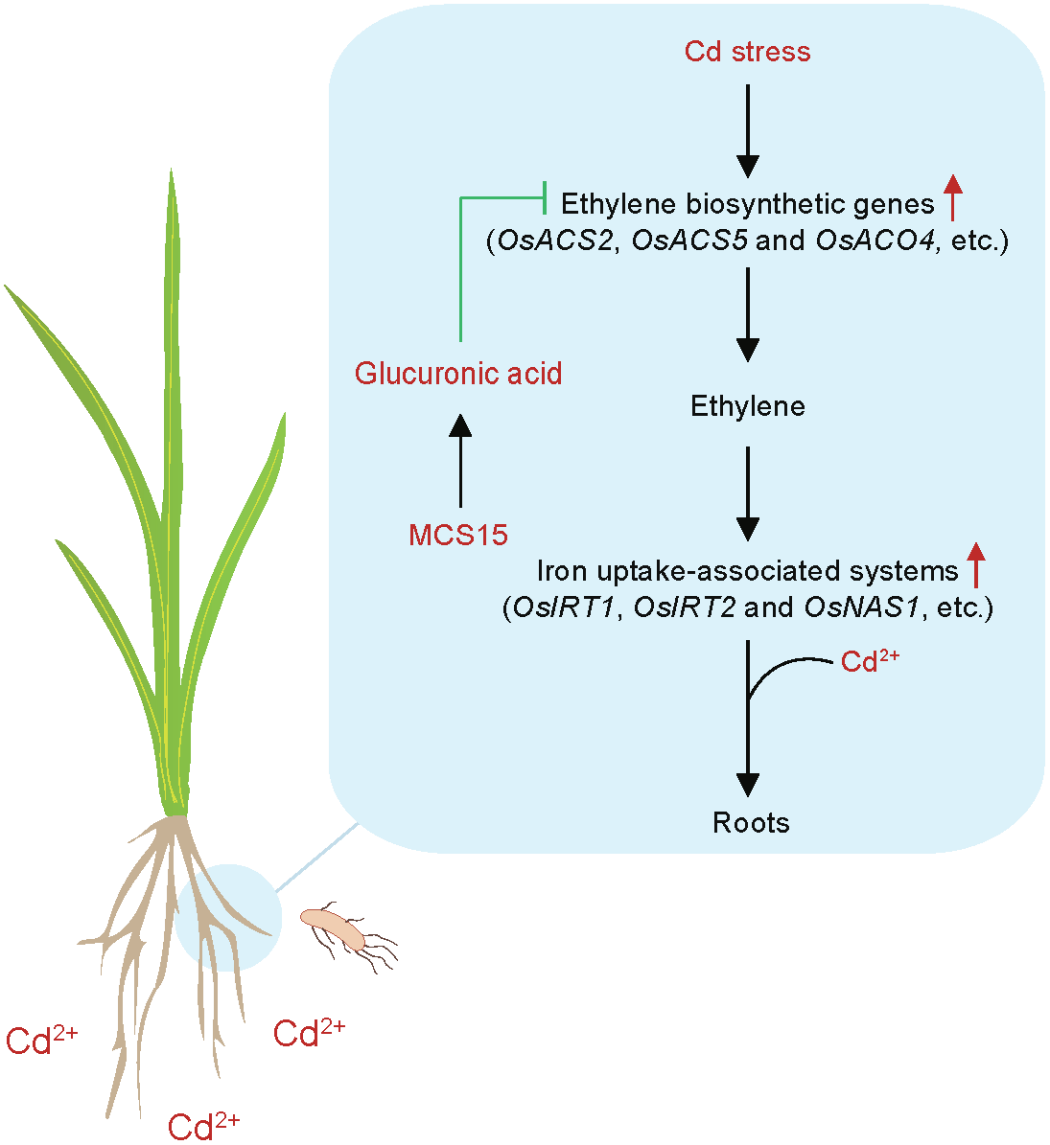
A model for illustrating the endophyte *Pseudomonas* sp. MCS15-induced Cd resistance in rice plants. MCS15-secreted glucuronic acid inhibited the biosynthesis of ethylene in the roots and thus weakened the iron uptake-related systems, which contributed to inhibition of Cd uptake.

Although diverse endophytic bacteria have been demonstrated to improve the Cd resistance in plants, the molecular mechanisms of the endophyte-induced stress adaptation remain rarely clear. In this study, a model was proposed for the MCS15-induced Cd resistance in rice plants (Figure 8). Cd stress stimulated the expression of ethylene biosynthetic genes, such as *OsACS2*, *OsACS5,* and *OsACO4*, and thus increased the production of ethylene in rice roots. The increased ethylene levels in the roots further led to the enhanced transcription of iron uptake-related genes, such as *OsIRT1*, *OsIRT2,* and *OsNAS1*, which contributed to promoting the Cd uptake in plants. However, the inoculation with MCS15 markedly increased the levels of glucuronic acid in the roots and thus inhibited the expression of ethylene biosynthetic genes, which contributed to reduction of ethylene production. This further weakened the iron uptake-related systems in the MCS15-inoculated roots. We further revealed that the repressed host Cd uptake by MCS15 was closely related to the glucuronic acid-mediated inhibition of ethylene biosynthesis. Our findings provided important evidence that the application of glucuronic acid-producing bacteria to reduce host ethylene production can be applied to prevent the Cd accumulation in plants grown under Cd-polluted conditions.

## Data Availability Statement

The datasets presented in this study can be found in online repositories.The names of the repository/repositories and accession number(s) can be found at: NCBI SRA database (PRJNA807004) and MetaboLights (MTBLS4322).

## Author Contributions

JX and RW: conceptualization and supervision. JX and FS: investigation and formal analysis. RW: funding acquisition. LQ and FS: experiments. LQ: analysis of results. LQ, FS, and RW: writing original draft. RW and LQ: review and editing. All authors contributed to the article and approved the submitted version.

## Funding

This work was supported by the Major Science and Technology Project of Anhui Province (201903a06020023) and Undergraduate Innovation and Entrepreneurship Training Program of Anhui Province (202010879003).

## Conflict of Interest

JX was employed by Anhui Shengnong Agricultural Group Co., Ltd.

The remaining authors declare that the research was conducted in the absence of any commercial or financial relationships that could be construed as a potential conflict of interest.

## Publisher’s Note

All claims expressed in this article are solely those of the authors and do not necessarily represent those of their affiliated organizations, or those of the publisher, the editors and the reviewers. Any product that may be evaluated in this article, or claim that may be made by its manufacturer, is not guaranteed or endorsed by the publisher.
