# Supplementary material for: A Glucuronic Acid-Producing Endophyte Pseudomonas sp. MCS15 Reduces Cadmium Uptake in Rice by Inhibition of Ethylene Biosynthesis
Source: Front Plant Sci. 2022 Apr 14;13:876545. doi: 10.3389/fpls.2022.876545 (PMC9047996; doi:10.3389/fpls.2022.876545)
Supplement: Supplementary file 1 [file Data_Sheet_1.ZIP › Supplementary_Material.docx]

Supplementary Material

**Figure S1.** Colonization of MCS15 in rice roots. 10-d-old rice seedlings were treated with 0 or 80 μM CdCl_2_ in the presence or absence of MCS15 for two weeks. Then, the colonization of MCS15 in rice roots was quantified by qPCR. Different letters indicate significant differences using Tukey’s multiple comparison test at *p* < 0.05. Error bars show ± SD from n = 3 biological repeats.

**Table S1.**  Primers used in this study for qPCR analyses

**Table S2.** Up- and down-regulated DEGs between the non-inoculated and MCS15-inoculated plants.

**Table S3.** Transcription profiles of genes involved in both the Fe uptake- and ethylene biosynthesis-related pathways.
