# Supplementary figures and images for: A Glucuronic Acid-Producing Endophyte Pseudomonas sp. MCS15 Reduces Cadmium Uptake in Rice by Inhibition of Ethylene Biosynthesis
Source: Front Plant Sci. 2022 Apr 14;13:876545. doi: 10.3389/fpls.2022.876545 (PMC9047996; doi:10.3389/fpls.2022.876545)

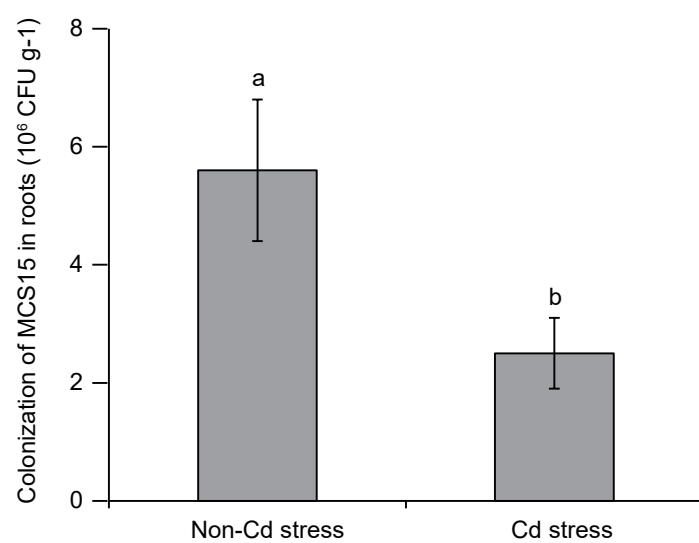

Supplement: Supplementary file 1 [file Data_Sheet_1.ZIP › Figure S1.pdf]
